# Dementia and Physical Activity (DAPA) - an exercise intervention to improve cognition in people with mild to moderate dementia: study protocol for a randomized controlled trial

**DOI:** 10.1186/s13063-016-1288-2

**Published:** 2016-03-25

**Authors:** Nicky Atherton, Chris Bridle, Deborah Brown, Helen Collins, Sukhdeep Dosanjh, Frances Griffiths, Susie Hennings, Kamran Khan, Ranjit Lall, Samantha Lyle, Rupert McShane, Dipesh Mistry, Vivien Nichols, Stavros Petrou, Bart Sheehan, Anne-Marie Slowther, Margaret Thorogood, Emma Withers, Peter Zeh, Sarah E. Lamb

**Affiliations:** Warwick Clinical Trials Unit, Warwick Medical School, University of Warwick, Gibbet Hill Road, Coventry, CV4 7AL UK; Lincoln Institute for Health, University of Lincoln, Brayford Pool, Lincoln, LN6 7TS UK; Nuffield Department of Orthopaedics, Rheumatology and Musculoskeletal Sciences, Kadoorie Centre, John Radcliffe Hospital, Headley Way, Oxford, OX3 9DU UK; John Radcliffe Hospital, Oxford University Hospitals, Oxford, OX3 9DU UK

**Keywords:** Dementia, Exercise, Randomised controlled trial

## Abstract

**Background:**

Dementia is more common in older than in younger people, and as a result of the ageing of the population in developed countries, it is becoming more prevalent. Drug treatments for dementia are limited, and the main support offered to people with dementia and their families is generally services to mitigate against loss of function. Physical exercise is a candidate non-pharmacological treatment for dementia.

**Methods/Design:**

DAPA is a randomised controlled trial funded by the National Institute for Health Research Health Technology Assessment programme to estimate the effect of a 4-month, moderate- to hard-intensity exercise training programme and subsequent advice to remain active, on cognition (primary outcome) at 12 months in people with mild to moderate dementia. Community-dwelling participants (with their carers where possible), who are able to walk 3 metres without human assistance, able to undertake an exercise programme and do not have any unstable or terminal illness are recruited. Participants are then randomised by an independent statistician using a computerised random number generator to usual care or exercise at a 2:1 ratio in favour of exercise. The exercise intervention comprises 29, 1-hour-long exercise classes, run twice weekly at suitable venues such as leisure centres, which include aerobic exercise (on static bikes) and resistance exercise (using weights). Goals for independent exercise are set while the classes are still running, and supported thereafter with phone calls.

The primary outcome is measured using ADAS-cog. Secondary outcome measures include behavioural symptoms, functional ability, quality of life and carer burden. Primary and secondary outcomes will be measured at baseline and at 6 and 12 months after randomisation, by researchers masked to participant randomisation in the participants’ own homes. An economic evaluation will be carried out in parallel to the RCT, as will a qualitative study capturing the experiences of participants, carers and staff delivering the intervention.

**Discussion:**

The DAPA study will be the first large, randomised trial of the cognitive effects of exercise on people with dementia. The intervention is designed to be capable of being delivered within the constraints of NHS service provision, and the economic evaluation will allow assessment of its cost-effectiveness.

**Trial registration:**

DAPA was registered with the ISRCTN database on 29 July 2011, registration number ISRCTN32612072.

## Background

Dementia is a syndrome characterised by acquired, progressive deterioration in memory, general cognitive function, self-care and personality. Probable dementia according to the *Diagnostic and Statistical Manual, 4th Edition* (*DSM IV*) criteria is defined by:

- Memory impairment with cognitive disturbance in at least one of the following domains: aphasia (language impairment), apraxia (motor impairment), agnosia (impairment of object recognition) or executive functioning (planning, sequencing, abstracting); and
- Functional decline: increasing impairment in functional ability (social, occupational, personal/self-care) related to cognitive deficits.

About 60 % of cases of dementia in developed countries are caused by Alzheimer’s disease, and about 20 % are vascular dementia [1], while mixed Alzheimer/vascular dementia and dementia with Lewy bodies are the other common causes. Dementia affects older people to a much greater extent than younger people. The prevalence of dementia in developed countries is under 1 % for people aged 65–69 years, rising to about 35 % in people aged 95–99 years, and prevalence doubles in successive 5-year age groups within the age range 65–99 years [2]. Prevalence is similar in men and women [2].

Treatments for dementia recommended by the National Institute for Health and Care Excellence (NICE) [3] are cholinesterase inhibitors (donepezil, galantamine and rivastigmine), but only for mild to moderate Alzheimer’s disease (not for vascular dementia). Memantine is supported by NICE for limited use in moderate to severe dementia, or in patients unable to tolerate cholinesterase inhibitors [3]. Many people with mild to moderate dementia and their families require additional services, mainly to mitigate functional loss, such as carer training, home carers, day care, respite admissions, sitting services and carer support services.

Physical exercise is a candidate non-pharmacological treatment for dementia. The most recent Cochrane review of physical activity for people with dementia [4] included 17 studies, nine of which assessed the effect of exercise on cognition. The authors concluded that there was encouraging evidence for the effectiveness of physical activity in improving cognition and other outcomes in people with dementia. However, these studies suffer from having small sample sizes, short-term follow-up and other methodological problems. There is a need for large, randomised trials of exercise in people with dementia.

### Objectives

The primary objective is to undertake a definitive randomised controlled trial (RCT) to estimate the effect of a 4-month, moderate- to hard-intensity exercise training programme and subsequent advice to remain active on (1) global cognition (primary outcome) and (2) function, behaviour, health-related quality of life and carer burden (secondary outcomes) in community-dwelling people with mild to moderate dementia.

Other objectives are to conduct a parallel economic evaluation from a National Health Service (NHS) and personal social services perspective and from a societal perspective, with a view to estimating the cost-effectiveness of the programme, and to complete a qualitative study alongside the trial.

## Methods/Design

### Trial design

**Fig. 1:**
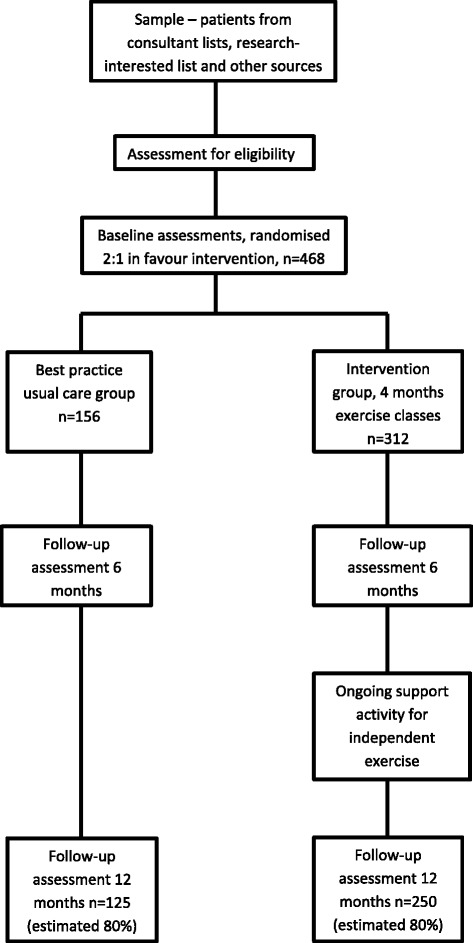
Flowchart of study design

We have designed a multi-centred, randomised parallel group trial with an economic evaluation to compare a 4-month, supervised, moderate- to hard-intensity exercise training regime with follow-on behavioural support for long-term physical activity change, in comparison to best practice usual care. The study design is outlined in Fig. 1. Participants are randomised in unbalanced allocation prior to entry into the study, then followed up for 12 months. Carers are recruited to provide an additional data set. Participants, carers, and treating therapists not are masked, but all baseline and follow-up assessments are undertaken by masked assessors. The analysis will be undertaken by statisticians who are masked to the allocation code. More details are available in the latest version of the Dementia and Physical Activity (DAPA) protocol, version 2.0, dated 7 July 2014.

### Study oversight

The overall supervision of the trial will be carried out by the Trial Steering Committee (TSC). This committee comprises the Chief Investigator, trial manager, statistician, and four independent members (including the committee chair). The TSC will monitor trial progress and conduct, and provide expert advice. In addition, an independent Data Monitoring and Ethics committee (DMEC) comprising three independent members, will review the progress of the trial and review safety data as it emerges. The DMEC will consist of independent experts with relevant clinical research and statistical experience. During the period of recruitment into the trial, reports of the accumulating data will be supplied, in strict confidence, to the DMEC, along with any other reports that the committee may request. There is no formal interim analysis proposed.

All substantial amendments to the protocol will be submitted to the ethics committee for approval. All non-substantial amendments will be submitted to the ethics committee for notification purposes. In addition, all substantial and non-substantial amendments to the protocol will be notified on a regular basis to the Trial Steering Committee, Data Monitoring Committee and Trial Management Committee. Where appropriate, and of operational purpose, the amendments will be notified to the Principal Investigators and other relevant trial and clinical staff. Trial registers will be informed of substantial amendments.

The trial sponsor is the University of Warwick. The sponsor has no role in the trial design; collection, management, analysis or interpretation of data; writing of reports and submission for publication.

### Ethics, ethical issues and arrangements for consent

Ethics approval for the DAPA trial was given by the National Research Ethics Committee (South West – Frenchay) REC number 11/SW/0232, on 19 January 2012. People with mild to moderate dementia may lack the necessary mental capacity to provide fully informed consent. All potential participants are assessed carefully for their capacity to consent by trained healthcare professionals. Where potential participants are assessed as having capacity, informed consent is obtained from the individual. Where potential participants are assessed as lacking capacity, in compliance with the Mental Capacity Act 2005 and best practice, agreement to participate is still sought from these individuals. In addition, we obtain advice from the primary carer/personal consultee, on whether the person who lacks capacity should take part in the project, and what their past and present wishes and feelings would have been about taking part. For those participants unable to give informed consent, and for whom no personal consultee is available, we will seek a nominated consultee (e.g. healthcare professional) who is well-placed and prepared to act on behalf of the potential participant. Where a potential participant lacks capacity to provide informed consent, and a nominated consultee cannot be found, that person will not be recruited. Consent will be obtained from carers for their own participation in data collection and qualitative interviews.

### Setting

We will recruit community-dwelling people with mild to moderate dementia and, where available, their carers. Potential participants will be identified from a number of sources: secondary and primary care clinics, registers of people with dementia who have expressed an interest in participating in research (called research interested lists) and community dementia resources e.g. dementia cafes.

We aim to recruit a sample to provide estimates of effect of the exercise intervention that are generalizable to the range of settings and populations served by NHS England. In terms of geographical spread, we will include both urban and more rural areas. NHS organisations included are: Sandwell and West Birmingham Clinical Commissioning Group (CCG), Telford CCG, Nene CCG, West Leicestershire CCG, Coventry and Warwickshire Partnership Trust, Oxford Health NHS Foundation Trust, Berkshire Healthcare NHS Foundation Trust, Worcestershire Health and Care Foundation Trust, Northamptonshire Healthcare NHS Foundation Trust, Leicestershire Partnership NHS Trust, Solent NHS Trust, Royal Devon and Exeter NHS Foundation Trust, Greater Manchester West Mental Health NHS Foundation Trust, Black Country Partnership NHS Foundation Trust, Coventry and Rugby CCG, South Warwickshire CCG, Warwickshire North CCG, South Worcestershire CCG, Redditch and Bromsgrove CCG, 2gether NHS Foundation Trust and North East London NHS Foundation Trust.

### Approach

Potential participants will be approached from four sources:

1. NHS secondary care memory clinics and services. Research assistants will identify potential participants using electronic or case record searches to screen for eligibility. The lists are reviewed by a clinician involved in the clinical care of the participant, and exclusions checked. Potential participants and their carers will then be approached either face-to-face at clinic or by letter. In either instance, the initial approach is made by the clinician known to the potential participant, and independent of the research team.
2. General Practice registers of people with dementia: GP practices will search their registers of patients to identify people with a diagnostic code for dementia. In most practices this will be done by a search of databases using read codes or Quality Outcomes Framework codes. Each GP practice lead will approve the final list of names, and invitation letters will be sent.
3. Research network Participant Interested Databases (e.g. the Dementia and Neurodegenerative Diseases Network (DeNDRoN); Join Dementia Research): the Department of Health in the UK has funded a number of networks and projects to enable people with dementia to register their interest in participating in research and to enable a rapid and simple approach. Potential participants will be identified by a Research Nurse from these databases. Participants deemed as meeting the inclusion criteria will be contacted by telephone to explain the study, and if potentially eligible, sent an invitation letter.
4. Other dementia resources (e.g. Alzheimer cafes): we will approach community-based dementia resources directly to determine if they are willing to approach potential participants. If agreed by the service providers, a researcher from the team will visit the centres and explain the study to potential participants and their carers. If any participants/carers are interested, the researcher will assess their potential eligibility and contact their healthcare provider to confirm eligibility.

Regardless of the method of approach, once a participant has confirmed their willingness to participate they will be contacted by a member of the recruitment team (a registered nurse or allied health professional) to further assess eligibility and explain the study. All participants will receive written and verbal information, and be given a minimum of 48 h to decide whether they wish to join the trial. We will visit all the participants in their own homes to confirm consent in writing, undertake a baseline assessment and then register and randomise the participant.

### Inclusion and exclusion criteria

To be included participants must:

1. Have probable dementia according to *DSM IV*;
2. Have probable mild to moderate dementia (>10 on the standardised Mini-Mental State Examination (sMMSE) [5]);
3. Be able to participate in a structured exercise programme determined by:
  - be able to sit in a chair, and walk 10 ft without human assistance;
  - have no unstable medical conditions, e.g. unstable angina, or acute or terminal illness;
4. Live in the community, alone or with a friend, relative or carer, or in sheltered accommodation.

### Baseline assessment

The standardised Mini-Mental State Examination (sMMSE) [5] will be carried out immediately after consent is obtained, as a screen to ensure that the participant is eligible. We will then collect demographic and descriptive data on the participants including age, gender, marital status, ethnicity, educational attainment, employment status, and type of dementia (vascular, Alzheimer’s or mixed). In addition, we will collect the pre-randomisation (baseline) values for the outcomes to be collected during the follow-up phase of the trial (see Table 1).

**Table 1:** Outcome measures taken at baseline, 6-month and 12-month time points

	Domain	Measure	Data supplied by
Primary	Cognition	Alzheimer’s Disease Assessment Scale-cognitive subscale (ADAS-cog) [6]	Participant (rating self)
Secondary	Function	Bristol Activities of Daily Living scale [8]	Carer (rating participant)
	Health-related quality of life	Euro-QoL (EQ-5D) [10]	Participant (rating self)
		Euro-Qol (EQ-5D)	Carer (rating self)
		Euro-Qol (EQ-5D) proxy	Carer (rating participant)
	Dementia quality of life	Quality of Life in Alzheimer’s Disease (QoL-AD) [9]	Participant (rating self)
		Quality of Life in Alzheimer’s Disease (QoL-AD) proxy	Carer (rating participant)
	Behavioural symptoms	Neuropsychiatric Inventory (NPI) [11]	Carer (rating participant)
	Carer burden	Zarit Burden Interview (ZBI) [12]	Carer (rating self)
	Health and social care usage to inform the health economics analysis	Client Services Receipt Inventory (CSRI) [13]	Participant with carer where possible (rating participant)

### Randomisation/masking

The unit of randomisation is the individual patient. Randomisation will be stratified by site and dementia severity (moderate or mild). Dementia severity is defined as moderate for those with an sMMSE >10 but <20, and as mild dementia for those with an sMMSE ≥20. Participants will be randomised to (1) usual practice care, or (2) usual practice care plus exercise intervention. Randomisation will be 2:1 in favour of the intervention group, to allow exercise groups to be assembled in a shorter period of time. As a result, participants are less likely to withdraw between randomisation and the exercise classes commencing, are not kept waiting excessively long periods between randomisation and treatment provision, and the baseline measures are made close to the exercise classes commencing.

The random allocation sequence will be generated by an independent statistician using a computerised random number generator, and implemented by a central telephone registration and randomisation service at the Warwick Clinical Trials Unit (WCTU).

Research clinicians (registered nurses or allied health professionals) will register participants after obtaining consent, confirming eligibility and undertaking a baseline assessment. Once registered, the allocation will be generated, and the WCTU DAPA trial team will inform the participant of their allocation and make arrangements for treatment referral. Neither intervention providers nor participants can be masked to treatment allocation. If a research clinician becomes unmasked then follow-up assessments will be conducted by different research workers, and all study personnel involved with data entry, follow-up assessments, management and analysis will be masked until the final analysis is complete.

### Choice of outcome measures

The primary outcome will be the global cognition score of the Alzheimer’s Disease Assessment Scale-cognitive subscale (ADAS-cog) [6], measured at 12-month follow-up. This instrument has 11 standard items, and additionally the maze and number cancellation optional items, which will be reported separately. It takes about 30–40 minutes to administer. The score ranges from 0 to 70, with higher scores indicating greater impairment. Cognitive deficits are central to dementia and widely understood as the most important treatment target. In the original study protocol, standardised Mini-Mental State Examination (sMMSE) [5] was the measure of cognition used. It was replaced by ADAS-cog on the recommendation of the TSC and DMEC because the latter is widely considered the gold standard primary outcome in treatment trials for dementia, with relatively well-established treatment effect sizes [7], and has superior sensitivity to change and hence is a more efficient instrument from the point of view of experimental design.

Secondary outcomes have been chosen to reflect the broad impact of dementia: on function, on behaviour, on quality of life, on economic costs, and on the carer. Where possible, we have chosen instruments which are dementia-specific, well-validated and not excessively burdensome. Secondary outcomes are detailed in Table 1.

We will use the Bristol Activities of Daily Living scale (BADL) [8]. This carer-rated instrument is dementia-specific and covers 20 activities of daily living. It is sensitive to change and widely used in clinical trials. Quality of life is widely considered important in dementia. We will collect data using the Quality of Life in Alzheimer’s Disease (QoL-AD) [9], a 13-item dementia-specific scale which can be completed by a carer or participant (both will be collected during the DAPA trial). We will also collect the EuroQol EQ-5D [10], a 5-dimension generic (i.e. not dementia-specific) measure of health-related quality of life. This will be collected from both the participant’s and the carer’s perspective. EuroQol EQ-5D allows a calculation of health utilities for application in economic evaluations.

We will use the Neuropsychiatric Inventory (NPI) [11]. This scale assesses 12 dimensions of possible behavioural disturbance in dementia, including important predictors of care breakdown like depression and agitation, using a screening strategy to save time. We will collect data using the Zarit Burden Inventory (ZBI) [12]. This short scale is the most commonly used dementia-specific carer burden tool. We will collect cost data using the Client Services Receipt Inventory (CSRI) [13]. This detailed questionnaire is used with a carer and covers all social, health care, medication use and out of pocket expenses. It will be adapted for DAPA.

For carers, we will record carer age, gender, ethnicity, and details about the relationship they have with the person with dementia and how much care they provide. They will be asked to complete the Zarit Burden Interview [12], which measures carer burden, and the EuroQol EQ-5D [10] measuring their own health-related quality of life. They will be asked to complete several measures regarding the person with dementia whom they care for: Bristol Activities of Daily Living (BADL) scale [8], Proxy QoL-AD [9], the number of falls and fractures the person has had, and the NPI [11].

Additionally, at the 6-month and 12-month follow-up interviews, the carer will be asked “How much benefit have you gained from being in the DAPA trial?” and “How much has the dementia of the person you care for changed in the past 6 months?” The CSRI is usually asked of the carer about the participant at this time point, but a person with no carer can give as much information as they are able.

These outcomes are all among those recommended by a consensus recommendation of outcome scales for non-drug interventional studies in dementia [14].

### Training and quality assurance in the research protocol

We will train all staff involved in recruitment and baseline assessments during a 1-day face-to-face training session, supplemented by a detailed operational Recruitment Manual. The ADAS-cog is a measure which needs initial training, shadowing and practice over time to become proficient. Potential recruitment staff have differing experience of using this measure so training will be adapted to accommodate this. Those with a working knowledge of the measure will attend a data collection training day which will include: rating a video of an ADAS-cog administration on a simulated patient, discussion around individual items and performing an ADAS-cog simulation with a trainer to assess practical competency. Based on competency, we will decide whether the rater requires further shadowing and training. Inexperienced raters will have an initial introduction to the measure, shadow experienced raters and practice until they are ready to undertake the training day. They will also be required to pass a competency test. This day will also include training for the sMMSE screening tool and other outcome measures.

Quality control (QC) will include the observation of at least one baseline home visit per researcher to ensure recruitment and data collection processes are followed correctly. All questionnaire data will be sent to WCTU, where it will be checked on receipt for discrepancies and errors. We will provide feedback to the research nurses and therapists by email to improve standards. Further QC visits will be used to check source data collection and completion of paperwork as necessary.

### Study interventions

#### Usual care

All participants will receive usual care in compliance with the recommendation of the NICE clinical guidance, comprising information provision and limited social support. We recognise that the usual care interventions are variable across the country, and hence we will stratify the randomisation within centre to ensure these effects are randomly distributed. To account for any such differences in the control arm, the analyses will adjust for “centre” as a covariate. There will be no limit on co-interventions during the trial, medications and other treatments may be initiated, continued or discontinued at the discretion of the clinical team responsible for the care of the participants. We will collect data on treatments provided to both arms of the trial during the follow-up period, and describe these in the study reports. Exercise is not currently part of recommended usual care for people with dementia, but each study participant (in both the control and intervention groups) will be given one of two information sheets produced by the Department of Health, appropriate to their age group. The information sheets recommend physical activity levels for adults (aged 19–64) [15] and older adults (aged 65 and over) [16].

#### Experimental intervention

The rationale and development of the intervention has been described elsewhere [17]. In brief, the intervention is protocolised, with clear instructions on the selection, prescription and progression of exercise, behavioural elements and long-term physical activity promotion. The exercise intervention will be delivered in groups of around six to eight people, in suitable local venues such as leisure centres or gyms, led by a physiotherapist, and if needed an assistant. The physiotherapists and assistants will attend a one-and-a-half-day training programme which covers all elements of the intervention. Staff delivering the intervention will also be issued with a manual describing it in detail.

A pre-exercise assessment will estimate each participant’s fitness using a 6-minute walk test [18], and information on relevant co-morbidities and current exercise will be collected at this time. The classes will comprise aerobic exercise on static bicycles, and resistance exercises using dumbbells and weighted belts and jackets. The intensity of the aerobic exercise will be measured using the Borg scale of rating of perceived exertion (RPE) [19], modified for people with dementia [20]. The intensity of the exercise will increase, with the intention that by the final classes each participant should be working on the bicycle at low intensity (RPE = 2 [21]) for 5 minutes, and then at least at moderate (RPE = 4) intensity for 20 minutes, with part of this at hard (RPE = 6) intensity for those participants who are able; and in the resistance exercises performing ten repetitions with the ten repetition maximum weight, i.e. the heaviest weight that can be lifted ten times with good form. Each class will last approximately 1 hour, will be twice weekly with at least one free day between classes, and each participant will be scheduled to attend 29 classes over approximately 4 months.

The intervention group will be encouraged to begin some independent exercise whilst the exercise classes are still taking place, supported by the use of a local exercise opportunities booklet detailing local facilities and programmes, such as Exercise on Referral schemes and Walking for Health groups. These participants will also receive three phone calls and have one face-to-face meeting with the physiotherapist in the 8 months following the exercise classes, to support and encourage them to continue with independent exercise. Any participants who withdraw from the intervention, either during the exercise classes or the unsupervised exercise period, will be encouraged to remain in the study and complete the outcome measures at the 6-month and 12-month follow-up points.

Compliance with the intervention will be defined as participants who have attended 75 % of the scheduled exercise classes in the intervention arm.

### Intervention fidelity checks

A quality control (QC) visit will be made by the local intervention lead to the staff delivering the intervention in the first few weeks of their exercise classes. This is aimed principally at ensuring that the intervention is being delivered in a standardised manner, and that the physiotherapist and exercise assistant can demonstrate competency in all aspects of the intervention. The QC assessor will also provide clinical supervision and support to the intervention staff as needed.

Items assessed will include: correct adherence to the exercise procedures focusing on ensuring effective delivery of an adequate exercise dose; correct completion of all paperwork, especially the structured treatment forms which record the exercise intensity and duration (enabling accurate calculation of the exercise dose delivered); evidence of the use of a person-centred care approach with appropriate support given to participants; communication between the staff regarding participants’ progress and needs; and completion and return of adverse event documentation (where required). This visit will also provide support and advice to staff to enhance their clinical competencies and assist with smooth running of the exercise group. A second QC visit will be made after some of the formal behavioural support activities (such as goal setting) have been commenced. If possible, these activities will be observed, if this is not possible assessment will be via inspection of the documentation and discussion with the physiotherapist who carried out the activity, and corrective guidance will be given as needed. The QC assessor will also assess continued correct adherence to the exercise procedures, with an emphasis on the use of progressions and provision of adequate exercise challenges, and the correct completion of clinical records.

At the end of each of these visits feedback will be provided to the staff, and further visits arranged if problems are found in the QC assessment or if the staff need further support. A QC form will be completed and signed by both the assessor and the physiotherapist (provided that the latter agrees with its findings).

### Serious adverse event (SAE) and adverse event (AE) reporting

An adverse event (AE) is defined as any untoward medical occurrence in a participant which does not necessarily have a causal relationship with this treatment. These are most likely to be identified by the physiotherapist during the exercise sessions, from information at the sign-in, or after completion of the exercise sessions during support telephone calls or the face-to-face meeting.

As each participant will have had a pre-exercise assessment done, this will provide information on co-morbidities. The DAPA trial population is likely to include many participants over 70 years old and therefore common chronic diseases of older age will be anticipated, e.g. osteoarthritis. It is expected that participants will experience some uncomfortable effects of participation in the intervention, for example muscle or joint soreness in response to exercise. Provided these follow an expected pattern (e.g. as for delayed-onset muscle soreness), or need simple modifications to the exercise activity (e.g. changes to the bicycle seat height), or are non-serious exacerbations of existing medical conditions, they should not be considered as adverse events.

A serious adverse event (SAE) is an AE that fulfils one or more of the following criteria:

1. Results in death;
2. Is immediately life-threatening;
3. Requires hospitalisation or prolongation of existing hospitalisation;
4. Results in persistent or significant disability or incapacity;
5. Requires medical intervention to prevent one of the above.

SAEs to be reported in DAPA have been defined as those that occur as the result of an incident during, or within 2 hours of completing the exercise sessions or follow-on physical activities. SAEs will be reported to the Trial Co-ordinating Centre within 24 hours of the physiotherapist becoming aware of them. The Trial Co-ordinating Centre is responsible for reporting adverse events to the sponsor and ethics committee within required timelines.

The relationship of SAEs to trial treatment will be assessed by the Chief Investigator and this will be recorded on the SAE form. All SAEs will be recorded in the trial database, and where appropriate, reported to and reviewed by the DMEC throughout the trial, and will be followed up to resolution.

### Data management and data checking

All data will be managed within the framework of the Data Protection Act 1998 and the standard operating procedures of WCTU. Data will be entered onto a bespoke application and will be stored on a secure WCTU server with daily, weekly and monthly back-ups.

All case report forms (CRFs) and accompanying papers (excluding consent forms) are stored in a lockable cabinet at WCTU in individual, numbered participant files. The files are kept in numerical order and have restricted access. Consent forms will be stored separately as they contain identifiable information; the file will be kept in a different cabinet to the CRFs. Intervention forms are anonymised and stored in a lockable cabinet. All data will be checked for completeness and validity prior to data entry. Field researchers will be requested to check data for completeness prior to returning the CRFs to the DAPA trial team at WCTU. A further check will be made by an appropriate member of the trial team and queries clarified by the completing researcher prior to data entry. Data will not be checked and entered by the same trial personnel.

A detailed Data Management Plan has been written which provides full details of the management of the data including the tracking and collection of data from sites and participants, and guidance is provided for telephone contact with participants and carers.

### Statistics

#### Sample size

At the time of applying for research funding, our sample size was based on the standardised Mini-Mental Status Score [5] as the primary outcome measure. ISRCTN registration was completed on 29 July 2011 at the same time as the original ethical submission specifying the sMMSE as the primary outcome. During the process of developing the protocol, and with feedback from the TSC and DMEC, we changed the primary outcome to the ADAS-cog. We submitted this substantial amendment to the protocol for ethical approval, and this was granted on 17 July 2012. This was well in advance of the first randomisation to the trial, which was undertaken on 1 February 2013. The reasons for the change were that the ADAS-cog is acknowledged to be more sensitive in detecting cognitive change in individuals with mild to moderate dementia than a variety of other measures, including the sMMSE [22]. The ADAS-cog is able to characterise different domains of cognitive performance better than the other measures and has been recommended by an expert task force as the cognitive outcome measure of choice for research using participants with moderate cognitive impairment [22]. Unlike many other tests of cognition, the ADAS-cog has an expert-agreed between-group mean difference of about 2 to 2.5 points [23, 24], although smaller differences can be worthwhile [7]. This means that we can use a valid but more sensitive primary outcome measure. The potential sample size efficiency using the ADAS-cog was substantial.

We specified the sample size parameters as follows. We set the group difference of 2.45 ADAS-cog points. Initially, we set the pooled standard deviation at baseline at 6.3 ADAS-cog points based on a literature review [25]. We checked the value of the pooled standard deviation at baseline after 66 participants had been accrued into the trial, and revised this to 7.8 ADAS-cog points. There were no statistical or interim analyses undertaken and hence no inflation of the sample size for interim testing. We used 2:1 randomisation and made a small upward adjustment for this [26] (p.144 equation 3.2). This gives a sample size of 360 for alpha of 0.05 and a power of 80 %. Recognising that there might be clustering associated with the group format of the intervention, we inflated the sample using a design effect of 1.04 [intraclass correlation (ICC) = 0.01] in the intervention arm, assuming that there will be five participants per group (recognising that it may not be possible to achieve and retain eight recruits to each group), giving a sample size of 375. The sample size was further inflated to account for 20 % loss to follow-up, of which 10 % will be attributable to death. Thus a final sample size of 468 participants was set with 312 participants to be randomly allocated to the intervention arm and 156 participants to the control arm.

As the ADAS-cog takes up to 35 minutes longer to administer than the sMMSE, in order to reduce burden on the participants and contain the costs of the trial, we removed the Cornell Scale for Depression in Dementia (CSDD) from the protocol. The ISCTRN registration was updated with these amendments in December 2014.

#### Analyses

Data will be summarised and reported in accordance with Consolidated Standards of Reporting Trials (CONSORT) guidelines for randomised controlled trials [24], and we will use intention-to-treat analyses as the primary analysis. Hierarchical regression models will be used to estimate the treatment effects (with 95 % confidence intervals), as they allow us to account for the hierarchical data structure i.e. participants nested within exercise groups. We will estimate the group effects in the trial by entering the group variable into the hierarchical model as a random effect. If the group effects estimate is negligible, then multiple linear regression will be used to estimate treatment effects. The models will adjust for those covariates (age, gender, centre, and baseline ADAS-cog) which were considered to be most important in the light of clinical input. In addition we will estimate treatment effects over the 12-month time period using longitudinal models. Prespecified subgroup analyses looking at pre-randomisation variables of severity of cognitive impairment (sMMSE ≥20 and <20), type of dementia, physical performance and gender will be conducted using formal tests of interaction [23].

At the beginning of each exercise class, the physiotherapist sets aerobic exercise and resistance exercise targets specific to each participant, and then marks at the end of the class if the target was achieved. We refer to the targets set for each participant as the prescribed dose. Dose achieved will be described in two ways: (1) the percentage of the DAPA physiotherapist prescribed minutes of aerobic exercise at moderate intensity or harder that a participant achieved over the course of the DAPA exercise sessions, and (2) the percentage of the DAPA-prescribed resistance exercise dose a participant achieved over the course of the DAPA exercise sessions. For both definitions of dose, we will investigate the association between percentage prescribed dose achieved and the change in cognition by observing a simple scatter plot and then using linear regression models (unadjusted and adjusted). This will not equate to a dose–response analysis, as the dose has not been experimentally determined.

Complier average causal effect (CACE) analysis will be used to assess the effect of compliance with the intervention on the primary outcome [27]. The primary analysis will use participant-reported data. In the situations where we have collected proxy data routinely, we will undertake sensitivity analyses using these data.

The final anonymised data set will be accessible to all study members after data lock. The Chief Investigator will assume overall responsibility for the data report, and will have full access to the trial data set. There are no contractual agreements that limit access for investigators. The analysis plans described here will be finalised and approved by the Trial Steering Committee and Data Monitoring Committees prior to data lock.

### Economic evaluation

The most significant formal costs associated with dementia are the costs of social and long-term care [28]. Hospitalisation costs are also likely to occur, particularly related to falls and injuries [29]. The economic evaluation will therefore focus on the following major components of costs: NHS primary and secondary care, local authority care, the costs to other agencies or organisations, costs associated with institutional care and home care support. Intervention costs will reflect the costs necessary to implement the DAPA physical activity, including development and training, overheads, equipment, and staff-related expenses.

Two economic evaluations will be undertaken – first, *a within-trial evaluation*, and second, *a decision analytic-based cost-effectiveness model*. Both will be used to estimate the expected incremental cost per quality-adjusted life-year (QALY) gained for the DAPA interventions in comparison to best practice usual care. For both analyses, the primary perspective will be that of the UK NHS and personal social services. The potential impact of adopting a societal perspective on incremental cost-effectiveness ratios will be tested in sensitivity analyses using data on informal and indirect costs provided by carers at 6 and 12 months.

The within-trial analysis will compare the costs and outcomes between the study arms at the end of follow-up. The primary outcome measure will be the quality-adjusted life-year (QALY). Health-related quality of life (HRQoL) will be estimated using responses to the EuroQol EQ-5D [10] obtained from participants and their carers (participant self-report, carer proxy report and carer self-report). The primary analyses will be conducted using the EQ-5D responses obtained from the participants. Nevertheless, differences between participant and carer assessments of participant HRQoL will be acknowledged through sensitivity analyses. Utility weights will be taken from the UK General Population tariff for the EQ-5D [30, 31]. Unit costs will be taken from national databases including the NHS reference costs and the Personal Social Services Research Unit (PSSRU) costs of health and social care. Where national unit cost estimates are not available unit costs will be estimated using established accounting methods in consultation with NHS Trusts recruiting to the study. In line with current recommendations for best practice in economic analyses, costs and outcomes will be discounted at 3.5 % per annum [32]. Probabilistic sensitivity analysis will be undertaken using the non-parametric bootstrap. We will use the CSRI as a framework for resource data capture [33].

The decision analytic cost-effectiveness analysis model will use a lifetime time horizon to capture the full impact of any mortality and health-related quality of life differences on the long-term cost-effectiveness of the exercise intervention. It is likely that the model will have a semi-Markov structure to capture the time trend in the underlying risk of mortality, health-related quality of life, and costs of care. The methods for estimating health-related quality of life and utility, unit costs and discounting will be the same as for the within-trial analysis.

Probabilistic sensitivity analyses will be undertaken using Monte Carlo simulation techniques. The outputs reported from the analysis will be the same as for the within trial analysis.

### Qualitative study

We will conduct a qualitative study in parallel to the trial, the aim of which is to provide insight into participants’, carers’ and staff experiences of taking part in the experimental intervention. Semi-structured interviews with participants and carers and observations of the exercise classes will be carried out by a researcher trained and experienced in qualitative research methodologies. Observations will take place near the beginning, middle and towards the end of the supervised part of the intervention. We will conduct the interview study in two sections. The first section will interview participants as they experience the moderate to hard intensity training. We will recruit a second, separate sample and interview participants approximately 4 months after they have finished the supervised part of the intervention.

It is possible that some participants may have problems recalling their experiences. In those instances the focus of the interview will be on their current experiences of exercise. For those participants able to recall their experiences of the supervised part of the intervention interviews will seek to capture their experiences of taking part in both the supervised and unsupervised parts of the intervention. Current evidence suggests that doing research interviews with people with cognitive impairments presents a number of obstacles; these include short-term memory problems, difficulties in abstract thinking and expressive language, a lack of insight and awareness of their diagnosis, and damage to their sense-of-self related to their experiences of diagnosis and symptoms of dementia [34]. It is suggested that obstacles may be overcome by the interviewer adopting an attitude that assumes the person with dementia has something valuable to say, by listening carefully, accepting the interviewee as they are and with an openness to understand them. The deployment of strategies to minimise or overcome dementia-related obstacles include: taking a case-specific approach by taking the time to meet interviewees before the formal interview in order to build rapport and gauge expressive skills, being comfortable with long pauses and the expression of strong emotions, avoiding the use of complex concepts and adjusting the language to align with participants’ language, use of photographs as a memory aid and use of direct interview styles where expressive skills are very limited [34, 35]. The researcher undertaking the interviews has experience and training around these issues.

Interviews with carers will include a focus on participants’ and carers’ experiences of taking part in the supervised and unsupervised parts of the intervention and their experiences of living with dementia. We will interview participants until we feel confident that we have reached data saturation. Based on our previous experiences we anticipate that we will interview approximately 20 participants and their carers. Sampling will be purposive and will be aimed at reflecting the population of the intervention arm of the trial in relation to gender, ethnicity and social class. Pseudonyms will be used and any other identifying information will be anonymised to ensure participants’ confidentiality is maintained. Interview transcripts and field notes will be uploaded into NVivo (QSR International Pty Ltd, Melbourne, Australia), a computer program for qualitative data, to assist with data management and analysis.

Our approach to data analysis will be thematic and will broadly follow the principles of grounded theory [36]. This requires a constant moving back and forth within and across field notes and transcripts and starts from the first interview and continues throughout the data collection period and beyond. A thematic analysis involves identifying, describing, analysing, interpreting and reporting repeated patterns or themes and categories within participants’ transcripts using a deductive and inductive approach. A theme captures important meanings across participants in relation to the research question and represents some level of patterned response or meaning across a data set and the relevant research literature [37]. Transcripts will be analysed by the researcher carrying out the interviews and, to ensure rigorous analysis, a second senior researcher will independently analyse approximately 20 % of the transcripts [37]. These two researchers will meet to discuss the differences and similarities between the transcripts and their own interpretations of them will be explored. Discussion of the analytic process and its findings will be shared within the wider research team thereby enabling an opportunity for critical reflection and further analytic refinement. Disagreements regarding the identification, description, analysis or interpretation of themes will be resolved through discussion and if necessary further analysis.

## Discussion

Dementia becomes increasingly prevalent with increasing age. As the population in developed countries ages, it is probable that the number of people in that population with dementia will increase. Treatment options for people with mild to moderate dementia are limited, and physical activity or exercise has been shown to have some effects on cognition.

The DAPA study will be the first large, randomised trial of the cognitive effects of exercise on people with dementia. The intervention is designed to be capable of being delivered within the constraints of NHS service provision, and the economic evaluation will allow assessment of its cost-effectiveness. Results will be published in an appropriate peer-reviewed journal.

### Trial status

At the time of submission for publication, the DAPA trial was still recruiting participants.

## Abbreviations

ADAS-cog: Alzheimer’s Disease Assessment Scale, cognitive subscale
AE: adverse event
BADL: Bristol Activities of Daily Living scale
CACE: complier average causal effect
CCG: Clinical Commissioning Group (of the NHS)
CONSORT: Consolidated Standards of Reporting Trials
CRF: case report form
CSDD: Cornell Scale for Depression in Dementia
CSRI: Client Services Receipt Inventory
DAPA: Dementia and Physical Activity trial
DeNDRoN: Dementia and Neurodegenerative Diseases Network
DMEC: Data Monitoring and Ethics Committee
DSM IV: Diagnostic and Statistical Manual, 4th Edition
GP: General Practitioner
HRQoL: health-related quality of life
ICC: intraclass correlation
NHS: National Health Service (of the UK)
NICE: National Institute for Health and Care Excellence
NPI: Neuropsychiatric Inventory
PSSRU: Personal Social Services Research Unit
QALY: quality-adjusted life-year
QC: quality control
QoL-AD: Quality of Life in Alzheimer’s Disease
RCT: randomised controlled trial
RPE: rating of perceived exertion
SAE: serious adverse event
sMMSE: standardised Mini-Mental State Examination
TSC: Trial Steering Committee
WCTU: Warwick Clinical Trials Unit
ZBI: Zarit Burden Interview
